# Supplementary material for: Plant-Derived Substances in the Fight Against Infections Caused by Candida Species
Source: Int J Mol Sci. 2020 Aug 25;21(17):6131. doi: 10.3390/ijms21176131 (PMC7504544; doi:10.3390/ijms21176131)
Supplement: Supplementary file 1 [file ijms-21-06131-s001.pdf]

Table S1. Antifungal activity of plant extracts on *Candida* spp. Diverse plant extracts have been tested for growth inhibition of *Candida* spp., both wild (w) or isolated strains (i). Particular extracts demonstrated inhibition of biofilm formation (*).

**Plant**	**Extract**	**Compounds**	***Candida* spp.**	**References**
Adenocalymma alliacum	methanolic and ethanolic extracts		*C. albicans* i	[1]
Agastache rugosa (Fisch. and C. A. Mey.) Kuntze	essential oil		*C. albicans* w	[2]
Aralia continentalis	methanolic extract	phenolic compounds, flavonoids	*C. albicans* w	[3]
Camellia japonica	methanolic extract	phenolic compounds, flavonoids	*C. albicans* w	[3]
Chelidonium majus var. asiaticum	methanolic extract	phenolic compounds, flavonoids	*C. albicans* w	[3]
Chrysosplenium flagelliferum	methanolic extract	phenolic compounds, flavonoids	*C. albicans* w	[3]
Cinnamomum aromaticum (Cinnamomum cassia)	essential oil		*C. albicans* i<br>*C. glabrata* i<br>*C. krusei* i	[4]
Clinopodium nepeta (L.) Kuntze = *Calamintha nepeta* (L.) Sav	essential oil		*C. albicans* w	[5]
Convallaria keiskei	methanolic extract	phenolic compounds, flavonoids	*C. albicans* w	[3]
Corylopsis coreana	methanolic extract	phenolic compounds, flavonoids	*C. albicans* w	[3]
Curcuma longa	methanolic and ethanolic extracts		*C. albicans* i	[1]
Echinophora platybola	methanolic and ethanolic extracts		*C. albicans* i	[1]
Equisetum hyemale	ethanolic and aqueous extracts	chlorogenic acid, rosmarinic acid	*C. albicans* w,*<br>*C. kefyr* w<br>*C. geochares* w<br>*C. krusei* w	[6]
Eugenia caryophyllata Thumb.	essential oil		*C. albicans* i<br>*C. glabrata* i<br>*C. tropicalis* i	[7]

Euphorbia hirta	methanolic and ethanolic extracts		*C. albicans* [i]	[1]
Eurya emarginata	methanolic extract	phenolic compounds, flavonoids	*C. albicans* [w]	[3]
Firmiana simplex	methanolic extract	phenolic compounds, flavonoids	*C. albicans* [w]	[3]
Foeniculum vulgare Mill. var. dulce DC	essential oil		*C. albicans* [i]<br>*C. glabrata* [i]<br>*C. tropicalis* [i]	[7]
Geranium sibiricum	methanolic extract	phenolic compounds, flavonoids	*C. albicans* [w]	[3]
Hedera rhombea	methanolic extract	phenolic compounds, flavonoids	*C. albicans* [w]	[3]
Hippophae rhamnoides L.	methanolic extract	hydrolysable tannins, flavonoids, proanthocyanidins	*C. albicans* [w,*]<br>*C. glabrata* [i]	[8]
Hymenaea courbaril var. *courbaril*	essential oil	caryophyllene oxide, trans-caryophyllene	*C. albicans* [w]<br>*C. tropicalis* [w]<br>*C. parapsilosis* [w]<br>*C. glabrata* [w]<br>*C. krusei* [w]	[9]
Hyssopus officinalis L.	essential oil		*C. albicans* [i]<br>*C. tropicalis* [i]<br>C. *parapsilosis* [i]<br>*C. glabrata* [i]<br>*C. krusei* [i]	[10]
Ilex integra	methanolic extract	phenolic compounds, flavonoids	*C. albicans* [w]	[3]
Kirengeshoma koreana	methanolic extract	phenolic compounds, flavonoids	*C. albicans* [w]	[3]
Larrea cuneifolia	lactic acid-glucose-water extract	phenolics (caffeic acid, ferulic acid, quercetin, rosmarinic acid, nordihydroguaiaretic acid), alkaloids (piperine, theophylline)	*C. albicans* [i]	[11]
Larrea divaricata	lactic acid-glucose-water extract	phenolics (naringenin, caffeic acid, nordihydro—guaiaretic acid), alkaloids (piperine, theophylline)	*C. albicans* [i]	[11]
Lavandula angustifolia Mill.	essential oil	1,8-cineol, limonene, linalool, terpinen-4-ol	*C. albicans* [i,*]	[12]
Lavandula vera DC	essential oil		*C. albicans* [i]	[7]

			C. glabrata i<br>*C. tropicalis* i	
Lawsonia inermis	methanolic extract		*C. albicans* i	[1]
Lindera glauca	methanolic extract	phenolic compounds, flavonoids	*C. albicans* w	[3]
Lycoris radiate	methanolic extract	phenolic compounds, flavonoids	*C. albicans* w	[3]
Melaleuca alternifolia	essential oil	monoterpenes (terpinen-4-ol, γ-terpinene, α-terpinene, p-cymene, 1,8-cineol)	*C. krusei* i	[13]
Melissa officinalis L.	essential oil		*C. albicans* i<br>*C. glabrata* i<br>*C. tropicalis* i	[7]
Mentha longifolia	essential oil	thymol, carvacrol	*C. albicans* w,*	[14]
Mentha pulegium L.	essential oil		*C. albicans*<br>*C. krusei*<br>*C. parapsilosis*<br>*C. tropicalis*	[15]
Mentha requienii Bentham	essential oil		*C. albicans* w<br>*C. krusei* w	[16]
Mentha spicata L.	essential oil		*C. albicans*<br>*C. krusei*<br>*C. parapsilosis*<br>*C. tropicalis*	[15]
Mentha suaveolens Ehrh.	essential oil		*C. albicans* w	[17]
Mentha × piperita L.	essential oil		*C. albicans* w	[18]
	essential oil	menthol, menthone	*C. albicans* i<br>*C. krusei* i<br>*C. glabrata* i<br>*C. parapsilosis* i<br>*C. tropicalis* i<br>*C. valida* i<br>*C. lusitaniae* i<br>*C. norvegensis* i	[19]

Moluccella spinosa L.	essential oil		*C. albicans*	[20]
Myroxylon peruiferum	essential oil	spathulenol, α-pinene, caryophyllene oxide	*C. albicans* w<br>*C. tropicalis* w<br>*C. parapsilosis* w<br>*C. glabrata* w<br>*C. krusei* w	[9]
Ocimum basilicum L.	essential oil		*C. albicans* w	[21]
Ocimum sanctum L.	ethanolic and ethyl acetate extracts	methyl chavicol, linalool	*C. albicans* i	[22]
Oenothera laciniata	methanolic extract	phenolic compounds, flavonoids	*C. albicans* w	[3]
Origanum majorana L.	essential oil		*C. albicans* w	[21]
Origanum vulgare L.	lactic acid-glucose-water extract		*C. albicans* i	[11]
	essential oil		*C. albicans* w	[21]
Peganum harmala	ethanolic extract		*C. albicans* i,*	[23]
Pinus sylvestris	essential oil		*C. albicans* i<br>*C. glabrata* i<br>*C. tropicalis* i	[7]
Piper betle	ethanolic and ethyl acetate extracts	hydroxychavicol	*C. albicans* i	[22]
Pogostemon cablin (Blanco) Benth	essential oil		*C. albicans* w	[21]
Pogostemon parviflorus	methanolic and ethanolic extracts		*C. albicans* i	[1]
Pollia japonica	methanolic extract	phenolic compounds, flavonoids	*C. albicans* w	[3]
Polygonum sagittatum var. *sieboldi*	methanolic extract	phenolic compounds, flavonoids	*C. albicans* w	[3]
Premna microphylla Turcz.	essential oil		*C. albicans* w	[24]
Rhamnus prinoides	ethanolic extract		*C. albicans* w,*	[25]
Robinia pseudoacacia	methanolic extract	phenolic compounds, flavonoids	*C. albicans* w	[3]
Rosa rugosa	methanolic extract	phenolic compounds, flavonoids	*C. albicans* w	[3]
Rosmarinus officinalis L.	propylene glycol extract	1,8-cineole, camphor, α-pinene	*C. albicans* w,*	[26]
	essential oil		*C. albicans* w	[21]

Salvia fruticosa Miller	essential oil		*C. albicans* [w]	[27]
Salvia mirzayanii Rech. f. and Esfand	essential oil		*C. albicans* [w]<br>*C. glabrata* [w]<br>*C. krusei* [w]<br>*C. parapsilosis* [w]<br>*C. tropicalis* [w]	[28]
Salvia officinalis L.	essential oil		*C. albicans* [w]	[21]
	essential oil		*C. albicans* [i]<br>*C. glabrata* [i]<br>*C. tropicalis* [i]	[7]
Satureja hortensis L.	essential oil		*C. albicans*	[29]
Satureja thymbra L.	essential oil		*C. albicans* [w]	[27]
Securigera securidaca	ethanolic, butanolic and methanolic extracts		*C. albicans* [w]<br>*C. parapsilosis* [w]<br>*C. krusei* [w]	[30]
Sparganium stoloniferum	methanolic extract	phenolic compounds, flavonoids	*C. albicans* [w]	[3]
Swertia chirata	ethanolic extract		*C. albicans* [i]	[1]
Thapsia villosa	essential oil	monoterpene hydrocarbons, phenylpropanoids, limonene and methyleugenol	*C. albicans* [i]<br>*C. parapsilosis* [i]<br>*C. tropicalis* [i]<br>*C. krusei* [i]	[31]
Thuja orientalis	methanolic extract	phenolic compounds, flavonoids	*C. albicans* [w]	[3]
Thymbra spicata L.	essential oil		*C. albicans* [w]	[27]
Thymus bovei Benth.	essential oil		*C. albicans* [i]	[32]
Thymus schimperi Ronninger	essential oil		*C. albicans* [w]	[33]
Thymus vulgaris L.	essential oil		*C. albicans* [i]<br>*C. glabratea* [i]	[7]
	propylene glycol extract	thymol, carvacrol, linalool, geranoil, citral, tannins, organicacids, flavonoids, minerals, saponins, carotene, vitamin c	*C. albicans* [w,*]	[34]
	lactic acid-glucose-water extract		*C. albicans* [i]	[11]

	essential oil		*C. albicans* w	[21]
Vatairea macrocarpa	ethyl acetate extract	isoflavonoid: vatacarpan	*C. albicans*	[35]
Vismia guianensis	essential oil	humulene epoxide	*C. albicans* w<br>*C. tropicalis* w<br>*C. parapsilosis* w<br>*C. glabrata* w<br>*C. krusei* w	[9]
Vitex agnus-castus L.	essential oil		*C. albicans*<br>*C. glabrata*<br>*C. krusei*<br>*C. parapsilosis*<br>*C. tropicalis*	[36]
Vitex gardneriana	essential oil	sesquiterpenes (cis-calamenene, 6,9-guaiediene, caryophyllene oxide)	*C. albicans* w,*<br>*C. tropicalis* w,*	[37]
Wasabia koreana	methanolic extract	phenolic compounds, flavonoids	*C. albicans* w	[3]
Withania somnifera	methanolic and ethanolic extracts		*C. albicans* i	[1]
Zataria multiflora	essential oil	thymol, o-cymene, carvacrol	*C. albicans* w	[38]
Zingiber officinale	methanolic and ethanolic extracts		*C. albicans* i	[1]
Ziziphora tenuior L.	essential oil		*C. albicans*<br>*C. krusei*<br>*C. parapsilosis*<br>*C. tropicalis*	[39]

References

1. Samadi, F.M.; Suhail, S.; Sonam, M.; Sharma, N.; Singh, S.; Gupta, S.; Dobhal, A.; Pradhan, H. Antifungal efficacy of herbs. *J. Oral Biol. Craniofac. Res.* **2019**, *9*, 28–32, doi:10.1016/j.jobcr.2018.06.002.
2. Gong, H.; Li, S.; He, L.; Kasimu, R. Microscopic identification and in vitro activity of *Agastache rugosa* (Fisch. et Mey) from Xinjiang, China. *Bmc Complement. Altern. Med.* **2017**, *17*, 95, doi:10.1186/s12906-017-1605-7.
3. Choi, H.A.; Cheong, D.E.; Lim, H.D.; Kim, W.H.; Ham, M.H.; Oh, M.H.; Wu, Y.; Shin, H.J.; Kim, G.J. Antimicrobial and anti-biofilm activities of the methanol extracts of medicinal plants against dental pathogens *Streptococcus mutans* and *Candida albicans*. *J. Microbiol. Biotechnol.* **2017**, *27*, 1242–1248, doi:10.4014/jmb.1701.01026.

4. Szweda, P.; Gucwa, K.; Kurzyk, E.; Romanowska, E.; Dzierżanowska-Fangrat, K.; Zielińska Jurek, A.; Kuś, P.M.; Milewski, S. Essential oils, silver nanoparticles and propolis as alternative agents against fluconazole resistant *Candida albicans, Candida glabrata* and *Candida krusei* clinical isolates. *Indian J. Microbiol.* **2015**, *55*, 175–183, doi:10.1007/s12088-014-0508-2.
5. Božović, M.; Garzoli, S.; Sabatino, M.; Pepi, F.; Baldisserotto, A.; Andreotti, E.; Romagnoli, C.; Mai, A.; Manfredini, S.; Ragno, R. Essential Oil Extraction, Chemical Analysis and Anti-*Candida* Activity of *Calamintha nepeta* (L.) Savi subsp. *glandulosa* (Req.) Ball-New Approaches. *Molecules* **2017**, *22*, 203, doi:10.3390/molecules22020203.
6. Dos Santos Alves, C.F.; Bonez, P.C.; de Souza, M.E.; da Cruz, R.C.; Boligon, A.A.; Piana, M.; Brum, T.F.; Rossi, G.G.; Jesus, R.D.; Grando, T.H. Antimicrobial, antitrypanosomal and antibiofilm activity of *Equisetum hyemale*. *Microb. Pathog.* **2016**, *101*, 119–125, doi:10.1016/j.micpath.2016.11.008.
7. Mandras, N.; Nostro, A.; Roana, J.; Scalas, D.; Banche, G.; Ghisetti, V.; Del Re, S.; Fucale, G.; Cuffini, A.M.; Tullio, V. Liquid and vapour-phase antifungal activities of essential oils against *Candida albicans* and non-albicans *Candida*. *Bmc Complement. Altern. Med.* **2016**, *16*, 330, doi:10.1186/s12906-016-1316-5.
8. Sadowska, B.; Budzyńska, A.; Stochmal, A.; Żuchowski, J.; Różalska, B. Novel properties of *Hippophae rhamnoides* L. twig and leaf extracts—anti-virulence action and synergy with antifungals studied in vitro on *Candida* spp. model. *Microb. Pathog.* **2017**, *107*, 372–379, doi:10.1016/j.micpath.2017.04.020.
9. Costa, M.; Silva, A.; Silva, A.; Lima, V.; Bezerra-Silva, P.C.; Rocha, S.; Navarro, D.; Correia, M.; Napoleão, T.H.; Silva, M. Essential oils from leaves of medicinal plants of brazilian flora: Chemical composition and activity against *Candida* species. *Medicines* **2017**, *4*, 27, doi:10.3390/medicines4020027.
10. Hristova, Y.; Wanner, J.; Jirovetz, L.; Stappen, I.; Iliev, I.; Gochev, V. Chemical composition and antifungal activity of essential oil of *Hyssopus officinalis* L. from Bulgaria against clinical isolates of *Candida* species. *Biotechnol. Biotechnol. Equip.* **2015**, *29*, 592–601, doi:10.1080/13102818.2015.1020341.
11. Espino, M.; Solari, M.; Fernández, M.; Boiteux, J.; Gómez, M.R.; Silva, M.F. NADES-mediated folk plant extracts as novel antifungal agents against *Candida albicans*. *J. Pharm. Biomed. Anal.* **2019**, *167*, 15–20, doi:10.1016/j.jpba.2019.01.026.
12. Dolatabadi, S.; Salari, Z.; Mahboubi, M. Antifungal effects of *Ziziphora tenuior*, *Lavandula angustifolia*, *Cuminum cyminum* essential oils against clinical isolates of *Candida albicans* from women suffering from vulvovaginal candidiasis. *Infectio* **2019**, *23*, 222–226. doi:10.22354/in.v23i3.784.
13. Tullio, V.; Roana, J.; Scalas, D.; Mandras, N. Enhanced Killing of *Candida krusei* by Polymorphonuclear Leucocytes in the Presence of Subinhibitory Concentrations of *Melaleuca alternifolia* and "Mentha of Pancalieri" Essential Oils. *Molecules* **2019**, *24*, 3824, doi:10.3390/molecules24213824.
14. Pazarci, O.; Tutar, U.; Kilinc, S. Investigation of the antibiofilm effects of *Mentha longifolia* essential oil on titanium and stainless steel orthopedic implant surfaces. *Eurasian J. Med.* **2019**, *51*, 128–132, doi:10.5152/eurasianjmed.2019.18432.
15. Piras, A.; Porcedda, S.; Falconieri, D.; Maxia, A.; Gonçalves, M.; Cavaleiro, C.; Salgueiro, L. Antifungal activity of essential oil from *Mentha spicata* L. and *Mentha pulegium* L. growing wild in Sardinia island (Italy). *Nat. Prod. Res.* **2019**, 1–7. Advance online publication, doi:10.1080/14786419.2019.1610755.
16. Fancello, F.; Zara, S.; Petretto, G.L.; Chessa, M.; Addis, R.; Rourke, J.P.; Pintore, G. Essential oils from three species of *Mentha* harvested in Sardinia: Chemical characterization and evaluation of their biological activity. *Int. J. Food Prop.* **2017**, *20*, 1751–1761, doi:10.1080/10942912.2017.1354020.
17. Garzoli, S.; Pirolli, A.; Vavala, E.; Di Sotto, A.; Sartorelli, G.; Božović, M.; Angiolella, L.; Mazzanti, G.; Pepi, F.; Ragno, R. Multidisciplinary approach to determine the optimal time and period for extracting the essential oil from *Mentha suaveolens* Ehrh. *Molecules* **2015**, *20*, 9640–9655, doi:10.3390/molecules20069640.
18. Samber, N.; Khan, A.; Varma, A.; Manzoor, N. Synergistic anti-candidal activity and mode of action of *Mentha piperita* essential oil and its major components. *Pharm. Biol.* **2015**, *53*, 1496–1504, doi:10.3109/13880209.2014.989623.
19. Tullio, V.; Roana, J.; Scalas, D.; Mandras, N. Evaluation of the Antifungal Activity of *Mentha x piperita* (Lamiaceae) of Pancalieri (Turin, Italy) Essential oil and its synergistic interaction with azoles. *Molecules* **2019**, *24*, 3148, doi:10.3390/molecules24173148.

20. Casiglia, S.; Jemia, M.B.; Riccobono, L.; Bruno, M.; Scandolera, E.; Senatore, F. Chemical composition of the essential oil of *Moluccella spinosa* L. (Lamiaceae) collected wild in Sicily and its activity on microorganisms affecting historical textiles. *Nat. Prod. Res.* **2015**, *29*, 1201–1206, doi:10.1080/14786419.2014.995654.
21. Powers, C.N.; Osier, J.L.; McFeeters, R.L.; Brazell, C.B.; Olsen, E.L.; Moriarity, D.M.; Satyal, P.; Setzer, W.N. Antifungal and cytotoxic activities of sixty commercially-available essential oils. *Molecules* **2018**, *23*, 1549, doi:10.3390/molecules23071549.
22. Sivareddy, B.; Reginald, B.A.; Sireesha, D.; Samatha, M.; Reddy, K.H.; Subrahamanyam, G. Antifungal activity of solvent extracts of *Piper betle* and *Ocimum sanctum* Linn on *Candida albicans*: An *in vitro* comparative study. *J. Oral Maxillofac. Pathol.* **2019**, *23*, 333–337, doi:10.4103/jomfp.JOMFP_167_19.
23. Aboualigalehdari, E.; Sadeghifard, N.; Taherikalani, M.; Zargoush, Z.; Tahmasebi, Z.; Badakhsh, B.; Rostamzad, A.; Ghafourian, S.; Pakzad, I. Anti-biofilm properties of *Peganum harmala* against *Candida albicans*. *Osong Public Health Res. Perspect.* **2016**, *7*, 116–118, doi:10.1016/j.phrp.2015.12.010.
24. Zhang, H.Y.; Gao, Y.; Lai, P.X. Chemical composition, antioxidant, antimicrobial and cytotoxic activities of essential oil from *Premna microphylla* Turczaninow. *Molecules* **2017**, *22*, 381, doi:10.3390/molecules22030381.
25. Campbell, M.; Fathi, R.; Cheng, S.Y.; Ho, A.; Gilbert, E.S. *Rhamnus prinoides* (gesho) stem extract prevents co-culture biofilm formation by *Streptococcus mutans* and *Candida albicans*. *Lett. Appl. Microbiol.* **2020**, 10.1111/lam.13307, doi:10.1111/lam.13307.
26. de Oliveira, J.R.; de Jesus, D.; Figueira, L.W.; de Oliveira, F.E.; Pacheco Soares, C.; Camargo, S.E.; Jorge, A.O.; de Oliveira, L.D. Biological activities of *Rosmarinus officinalis* L. (rosemary) extract as analyzed in microorganisms and cells. *Exp. Biol. Med. (Maywood),* **2017**, *242*, 625–634, doi:10.1177/1535370216688571.
27. Khoury, M.; Stien, D.; Eparvier, V.; Ouaini, N.; El Beyrouthy, M. Report on the medicinal use of eleven *Lamiaceae* species in Lebanon and rationalization of their antimicrobial potential by examination of the chemical composition and antimicrobial activity of their essential oils. *Evid. Based Complement. Alternat. Med.* **2016**, 2547169, doi:10.1155/2016/2547169.
28. Zomorodian, K.; Moein, M.; Pakshir, K.; Karami, F.; Sabahi, Z. Chemical composition and antimicrobial activities of the essential oil from *Salvia mirzayanii* leaves. *J. Evid. Based Complement. Altern. Med.* **2017**, *22*, 770–776, doi:10.1177/2156587217717414.
29. Sharifzadeh, A.; Khosravi, A.R.; Ahmadian, S. Chemical composition and antifungal activity of *Satureja hortensis* L. essential oil against planktonic and biofilm growth of *Candida albicans* isolates from buccal lesions of HIV(+) individuals. *Microb. Pathog.* **2016**, *96*, 1–9, doi:10.1016/j.micpath.2016.04.014.
30. Raesi Vanani, A.; Mahdavinia, M.; Kalantari, H.; Khoshnood, S.; Shirani, M. Antifungal effect of the effect of *Securigera securidaca* L. vaginal gel on *Candida* species. *Curr. Med. Mycol.* **2019**, *5*, 31–35, doi:10.18502/cmm.5.3.1744.
31. Pinto, E.; Gonçalves, M.J.; Cavaleiro, C.; Salgueiro, L. Antifungal Activity of *Thapsia villosa* Essential Oil against *Candida, Cryptococcus*, *Malassezia*, *Aspergillus* and *Dermatophyte* Species. *Molecules* **2017**, *22*, 1595, doi:10.3390/molecules22101595.
32. Jaradat, N.; Adwan, L.; K'aibni, S.; Shraim, N.; Zaid, A.N. Chemical composition, anthelmintic, antibacterial and antioxidant effects of *Thymus bovei* essential oil. *Bmc Complement. Altern. Med.* **2016**, *16*, 418, doi:10.1186/s12906-016-1408-2.
33. Nasir, M.; Tafess, K.; Abate, D. Antimicrobial potential of the Ethiopian *Thymus schimperi* essential oil in comparison with others against certain fungal and bacterial species. *Bmc Complement. Altern. Med.* **2015**, *15*, 260, doi:10.1186/s12906-015-0784-3.
34. de Oliveira, J.R.; de Jesus Viegas, D.; Martins, A.; Carvalho, C.; Soares, C.P.;, Camargo, S.; Jorge, A.; de Oliveira, L.D. *Thymus vulgaris L.* extract has antimicrobial and anti-inflammatory effects in the absence of cytotoxicity and genotoxicity. *Arch. Oral Biol.* **2017**, *82*, 271–279, doi:10.1016/j.archoralbio.2017.06.031.
35. Santana, D.; Costa, R.; Araújo, R.; Paula, J.; Silveira, E.; Braz-Filho, R.; Espindola, L. Activity of Fabaceae species extracts against fungi and *Leishmania*: Vatacarpan as a novel potent anti-*Candida* agent. *Rev. Bras. Farmacogn.* **2015**, *25*, 401–406, doi:10.1016/j.bjp.2015.07.012.

36. Asdadi, A.; Hamdouch, A.; Oukacha, A.; Moutaj, R.; Gharby, S.; Harhar, H.; El Hadek, M.; Chebli, B.; Idrissi Hassani, L.M. Study on chemical analysis, antioxidant and in vitro antifungal activities of essential oil from wild *Vitex agnus*-castus L. seeds growing in area of Argan Tree of Morocco against clinical strains of *Candida* responsible for nosocomial infections. *J. Mycol. Med.* **2015**, *25*, e118–e127, doi:10.1016/j.mycmed.2015.10.005.
37. Vale, J.; Ribeiro, L.; Vasconcelos, M.A.; Sá-Firmino, N.C.; Pereira, A.L.; Nascimento, M.; Rodrigues, T.; Silva, P.; Sousa, K.C.; Silva, R. Chemical composition, antioxidant, antimicrobial and antibiofilm activities of *Vitex gardneriana* schauer leaves's essential oil. *Microb. Pathog.* **2019**, *135*, 103608, doi:10.1016/j.micpath.2019.103608.
38. Ardekani, N.T.; Khorram, M.; Zomorodian, K.; Yazdanpanah, S.; Veisi, H.; Veisi, H. Evaluation of electrospun poly (vinyl alcohol)-based nanofiber mats incorporated with *Zataria multiflora* essential oil as potential wound dressing. *Int. J. Biol. Macromol.* **2019**, *125*, 743–750, doi:10.1016/j.ijbiomac.2018.12.085.
39. Abu-Darwish, M.S.; Cabral, C.; Gonçalves, M.J.; Cavaleiro, C.; Cruz, M.T.; Paoli, M.; Tomi, F.; Efferth, T.; Salgueiro, L. *Ziziphora tenuior* L. essential oil from Dana Biosphere Reserve (Southern Jordan); Chemical characterization and assessment of biological activities. *J. Ethnopharmacol.* **2016**, *194*, 963–970, doi:10.1016/j.jep.2016.10.076.
